# New Spirometry Indices for Detecting Mild Airflow Obstruction

**DOI:** 10.1038/s41598-018-35930-2

**Published:** 2018-11-30

**Authors:** Surya P. Bhatt, Nirav R. Bhakta, Carla G. Wilson, Christopher B. Cooper, Igor Barjaktarevic, Sandeep Bodduluri, Young-il Kim, Michael Eberlein, Prescott G. Woodruff, Frank C. Sciurba, Peter J. Castaldi, MeiLan K. Han, Mark T. Dransfield, Arie Nakhmani

**Affiliations:** 1 Division of Pulmonary, Allergy and Critical Care Medicine and Lung Health Center, University of Alabama at Birmingham, Birmingham, AL 35294 USA; 2 UAB Lung Imaging Core, University of Alabama at Birmingham, Birmingham, AL 35294 USA; 3 Division of Pulmonary, Critical Care, Allergy and Sleep Medicine, University California San Francisco, San Francisco, CA 94143 USA; 4 Department of Biostatistics and Bioinformatics, National Jewish Health, Denver, CO 80206 USA; 5 Division of Pulmonary and Critical Care Medicine, David Geffen School of Medicine at the University California Los Angeles, Los Angeles, CA 90095 USA; 6 Department of Preventive Medicine, University of Alabama at Birmingham, Birmingham, AL 35294 USA; 7 Division of Pulmonary, Critical Care and Occupational Medicine, University of Iowa Hospital, Iowa City, IA 52242 USA; 8 Division of Pulmonary, Allergy and Critical Care Medicine, University of Pittsburgh, Pittsburgh, PA 15213 USA; 9 Channing Division of Network Medicine, Brigham and Women’s Hospital, Boston, MA 02115 USA; 10 Division of Pulmonary and Critical Care Medicine, University of Michigan, Ann Arbor, MI 48109 USA; 11 Department of Electrical and Computer Engineering, University of Alabama at Birmingham, Birmingham, AL 35294 USA

## Abstract

The diagnosis of chronic obstructive pulmonary disease (COPD) relies on demonstration of airflow obstruction. Traditional spirometric indices miss a number of subjects with respiratory symptoms or structural lung disease on imaging. We hypothesized that utilizing all data points on the expiratory spirometry curves to assess their shape will improve detection of mild airflow obstruction and structural lung disease. We analyzed spirometry data of 8307 participants enrolled in the COPDGene study, and derived metrics of airflow obstruction based on the shape on the volume-time (Parameter D), and flow-volume curves (Transition Point and Transition Distance). We tested associations of these parameters with CT measures of lung disease, respiratory morbidity, and mortality using regression analyses. There were significant correlations between FEV_1_/FVC with Parameter D (r = −0.83; p < 0.001), Transition Point (r = 0.69; p < 0.001), and Transition Distance (r = 0.50; p < 0.001). All metrics had significant associations with emphysema, small airway disease, dyspnea, and respiratory-quality of life (p < 0.001). The highest quartile for Parameter D was independently associated with all-cause mortality (adjusted HR 3.22,95% CI 2.42–4.27; p < 0.001) but a substantial number of participants in the highest quartile were categorized as GOLD 0 and 1 by traditional criteria (1.8% and 33.7%). Parameter D identified an additional 9.5% of participants with mild or non-recognized disease as abnormal with greater burden of structural lung disease compared with controls. The data points on the flow-volume and volume-time curves can be used to derive indices of airflow obstruction that identify additional subjects with disease who are deemed to be normal by traditional criteria.

## Introduction

The clinical diagnosis of chronic obstructive pulmonary disease (COPD) is based on the spirometric detection of airflow obstruction^1^. Approximately one-half of subjects without airflow obstruction by traditional spirometric criteria have substantial respiratory impairment or have structural lung disease on computed tomography (CT)^2,3^. These symptomatic smokers are also at increased risk of greater lung function decline and developing overt airflow obstruction on follow-up^4^. These findings point to the lack of sensitivity of traditional spirometric measures in detecting mild disease, and there is a need to develop novel metrics for the detection of mild airflow obstruction^5^.

The diagnosis of airflow obstruction currently relies on using fixed portions of the flow-volume curve that are not sensitive to detecting small airway disease. Previous attempts to detect mild small airways involvement have mostly relied on estimating the flow in the middle part of the flow-volume curve, examining the shape of a segment of the curve visually or through automated analyses^6–13^, or by examining the change in the angle of flow during forced exhalation^14,15^. Although these measures showed promising results, the results were limited by small sample sizes and lack of validation against structural lung disease. We hypothesized that mathematical modeling using all the data points on the expiratory curves to assess their shape would enable derivation of indices of airflow obstruction that improve detection of mild airflow obstruction as well as structural disease on CT.

## Methods

### Study population

We analyzed spirometry data of subjects enrolled in the Genetic Epidemiology of COPD (COPDGene) study, a large multicenter cohort that included current and former smokers aged 45–80 years; study details have been previously published^16^. All participants underwent extensive phenotyping with spirometry^17^, CT imaging, and assessment of respiratory morbidity using questionnaires and the six minute walk test (details in Supplement). Participants were followed every 6 months to ascertain vital status. All participants provided written informed consent prior to study enrollment and the COPDGene study was approved by the University of Alabama at Birmingham IRB for Human Use (F070712014) and the institutional review boards of all 21 participating centers (Details in Supplement). All assessments and analyses were performed in accordance with relevant guidelines and regulations.

### CT Metrics

Volumetric CT scans were obtained at maximal inspiration (total lung capacity, TLC) and end-tidal expiration (functional residual capacity, FRC). Emphysema and gas trapping were quantified using 3D Slicer software (www.airwayinspector.org), and Apollo Software (VIDA Diagnostics, Coralville, IA, USA) was used to measure airway dimensions^16^. Mild emphysema was quantified by using the percentage of lung volume at TLC with attenuation less than −910 Hounsfield Units (HU) (low attenuation area, %LAA910_insp_), and severe emphysema by %LAA < −950 HU^16^. We quantified gas trapping as the percentage of lung volume at end expiration with attenuation less than −856 HU^16^. We used Wall area percentage of segmental airways (Wall area pct) to quantify airway disease^16^. In addition, we used parametric response mapping to match inspiratory and expiratory images voxel-to-voxel, and calculated the percentage of non-emphysematous gas trapping, or functional small airways disease (PRM^fSAD^), a measure of small airways disease^18^.

### New Spirometry Metrics: Basis and Derivation

We used post-bronchodilator values for all analyses, and the effort with the highest value for the forced expiratory volume in the first second (FEV_1_) and the forced vital capacity (FVC) was selected for analyses per the American Thoracic Society (ATS) criteria^17,19^. Using advanced computational tools, we analyzed the individual data points in the flow-volume and volume-time curves (volume measurements collected every 60 msec and flow measurements every 30 ml), and developed the following metrics to quantify important transition points and contours in the expiratory curves.

#### Shape of the volume-time curve

**Figure 1:**
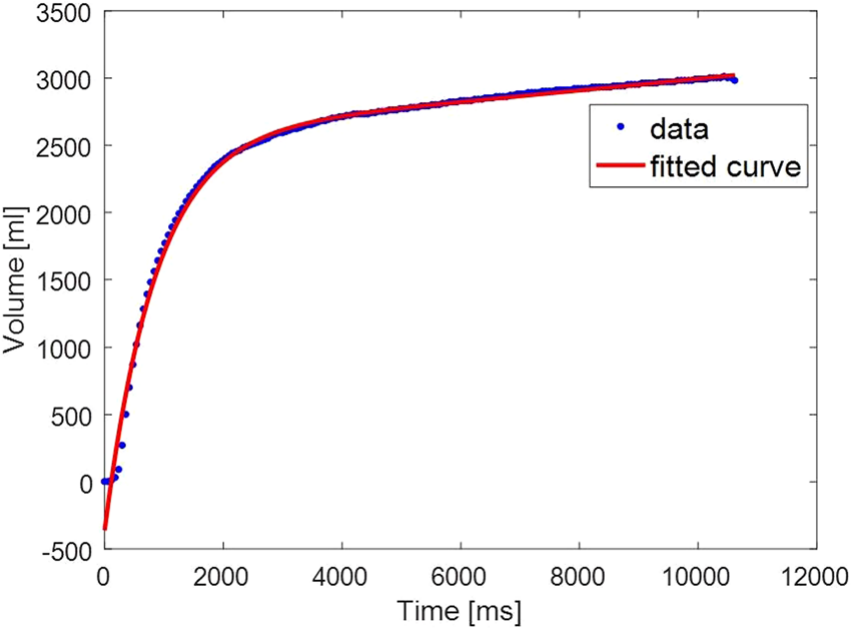
Model fitting of the Volume-time curve.

We used the Levenberg-Marquardt algorithm to fit the following model to the volume-time curve:*V*_*estimated*_ = *Ae*^*Bt*^ + *Ce*^*Dt*^ where *A*, *B*, *C*, *D* are the parameters found by the function fitting optimization process minimizing *J* = ||*V*_*measured*_ − *Ae*^*Bt*^ − *Ce*^*Dt*^|| cost function. To differentiate between the *Ae*^*Bt*^ and *Ce*^*Dt*^ terms, we always assume that *A* > 0 and *C* < 0. The first term, *Ae*^*Bt*^, represents the rising slope of volume increase closer to the end of the exhalation, and the second term, *Ce*^*Dt*^, describes the overall volume-time curve, where Parameter D describes the rate of volume increase. Figure 1 shows an example of such a function fit. The code for computing Parameter D is available in the Supplement.

#### Transition Point

**Figure 2:**
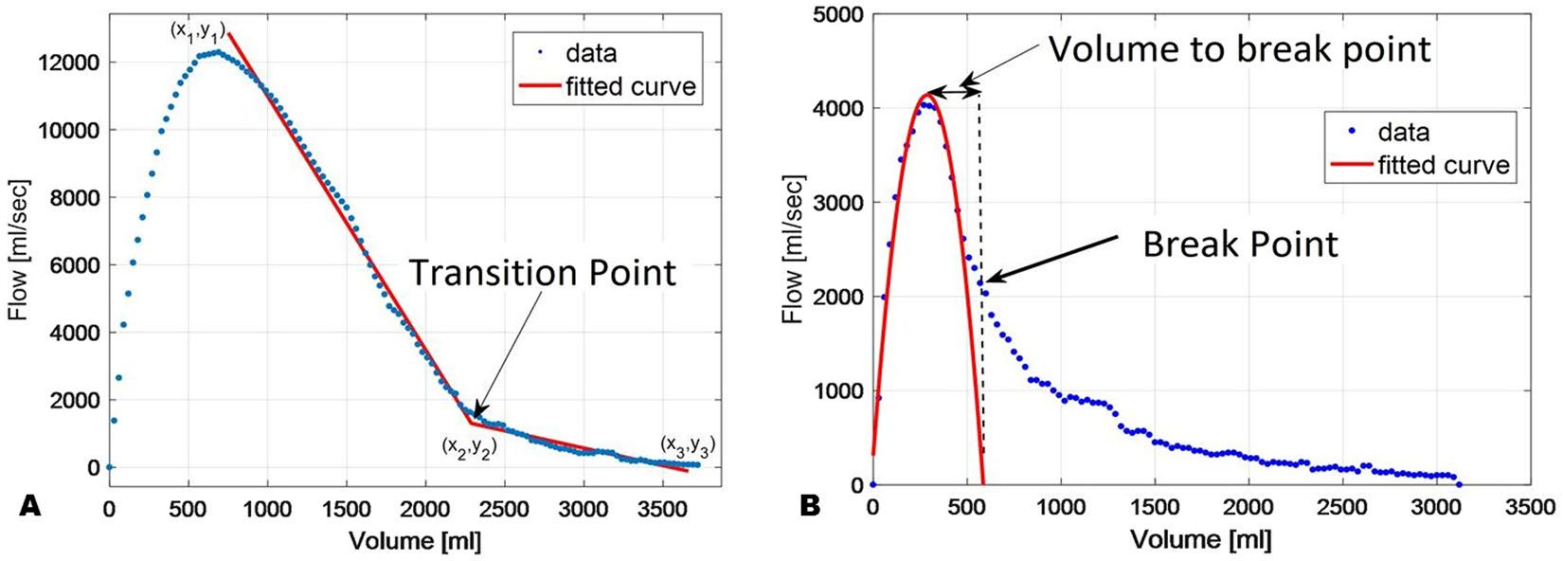
Computation of (**A**) the Transition Point, and (**B**). Computation of the breaking point and the change of volume from the maximum to the breaking point (Transition Distance).

We defined the transition point by fitting a piecewise function with two linear segments to the flow-volume curve, where the data before the peak expiratory flow is ignored (see Fig. 2A). A nonlinear least-squares algorithm was used to find the optimal fit parameters of the curve *(x*_1_*, y*_1_*),(x*_2_*, y*_2_*),(x*_3_*, y*_3_). The Transition Point is defined as *x*_2_.

#### Transition Distance

Given the Transition Point is not always readily apparent even with computational tools as the slopes may not fit on linear regression lines, we refined this process by fitting an inverted parabola around the peak point using a least squares minimization algorithm (see Fig. 2B). The breaking point between the parabola and the rest of the curve was defined as the latest sample that still provided goodness of fit of at least R^2^ > 0.96. The Transition Distance is the distance on the X-axis (in ml) from the peak of the fitted parabola to the breaking point (Fig. 2B**)**.

Details on the physiologic basis for the derivation of the new metrics are described in the Supplement.

### Case definitions

COPD was defined by FEV_1_/FVC < 0.70^20^. We excluded participants with Preserved Ratio Impaired SpiroMetry (PRISm, FEV_1_/FVC > 0.70 but FEV_1_ < 80% predicted) to avoid confounding by restrictive processes^21^. Using data from non-smokers, we calculated the 90^th^ percentile of normal for Parameter D (−0.104), and those greater than this threshold were deemed to have abnormal Parameter D. Those positive by both FEV_1_/FVC and Parameter D were defined as having COPD, and those negative by both criteria were deemed to have no airflow obstruction. Subjects positive by Parameter D but negative by FEV_1_/FVC were defined as additional cases detected by Parameter D (Discordant COPD). We repeated all comparisons with COPD defined by FEV_1_/FVC < 5^th^ percentile of predicted value for age, sex, race and height (lower limit of normal, LLN) as having COPD-LLN (Results in Supplement)^17,22^. Similarly, those <10th percentile of normal for Transition Point (17.0) and Transition Distance (30.0) were categorized to have airflow obstruction.

### Statistical analyses

Receiver operating characteristic (ROC) analyses measured the accuracy of the new spirometry indices in comparison with FEV_1_/FVC for identifying thresholds of structural lung disease on CT (5% severe emphysema and 5% functional small airway disease or fSAD). Generalized linear regression models were used to test associations between the new spirometry metrics and structural lung disease as well as respiratory morbidity indices. To assess performance of the new metrics in those with mild disease, we compared characteristics in those with GOLD stage 0 and 1 only, and tested concordance for diagnosis using FEV_1_/FVC < 0.70 (or < LLN) versus abnormal spirometry by new indices. Comparisons were made between those concordant and discordant for airflow obstruction by traditional and new spirometry indices with smokers concordant for not having airflow obstruction, using Analysis of Variance (ANOVA). With “normal” smokers as the reference group, adjusted odds ratios for CT measures of structural lung disease were estimated in each group. Cox proportional hazards were calculated for mortality for each higher quartile of Parameter D with the lowest quartile as the reference. Statistical significance was set at a two-sided alpha of 0.05. All analyses were performed using Statistical Package for the Social Sciences (SPSS 24.0, SPSS Inc., Chicago, IL, USA).

## Results

We first examined performance of the new metrics in 8307 participants with a full set of spirometry and CT data (see Supplemental Figure 7: CONSORT diagram). Mean age of participants was 60.0 (SD 9.1) years, and the cohort was comprised of 45.5% females and 31.1% African Americans. Parameter D, Transition point and Transition Distance could be calculated in 5532 (66.6%), 7960 (95.8%), and 7960 (95.8%) of expiratory curves. Parameter D ranged from −0.41 to 0.02, with more positive values indicating greater disease; Transition point ranged from 4.0 to 133.0 with lower values indicating worse disease; and Transition Distance ranged from 30.0 to 2220.0, with lower values indicating worse disease. Wherever Parameter D could not be automatically calculated, due to divergence of the function fitting algorithm and needing manual intervention, or the goodness of model fit was too low, the curves were discarded. The cohort encompassed a range of severity of airflow obstruction with 49.5%, 9.1%, 21.9%, 13.0%, and 6.5% with GOLD stages 0 through 4, respectively. Parameter D was progressively harder to calculate in more severe disease and could be calculated in 82%, 75%, 58%, 37% and 28%, respectively in participants with Global Initiative for Chronic Obstructive Lung Disease (GOLD) stages 0 through 4. As more severe disease is easily detected using traditional spirometry criteria, we focused on those with mild airflow obstruction in the second stage of analysis. In the overall cohort, there were significant correlations between FEV_1_/FVC and FEV_1_%predicted with Parameter D (r = −0.83; p < 0.001 and −0.66; p < 0.001, respectively), Transition Point (r = 0.69; p < 0.001 and 0.71; p < 0.001, respectively) and Transition Distance (r = 0.50; p < 0.001 and 0.52; p < 0.001, respectively).

### Association of New Metrics with Structural Lung Disease

#### Emphysema

Parameter D and FEV_1_/FVC were similar in accuracy for identifying >5% severe emphysema (%LAA < −950 HU) (c-statistic 0.83,95% CI 0.82–0.84; p < 0.001, and 0.83,95% CI 0.81–0.84; p < 0.001, respectively), whereas the c-statistic for Transition Point, Transition Distance, and FEV_1_%predicted were 0.71 (95% CI 0.70–0.73; p < 0.001), 0.68 (95% CI 0.66–0.69; p < 0.001), and 0.73 (95% CI 0.71–0.75; p < 0.001), respectively. Parameter D and FEV_1_/FVC also had comparable accuracy for identifying 10% severe emphysema (c-statistic 0.91,95%CI 0.89–0.92; p < 0.001, and 0.91,95% CI 0.90–0.93; p < 0.001, respectively). For 10% emphysema, Transition Point, Transition Distance and FEV_1_%predicted had improved accuracies with c-statistic of 0.81 (95% CI 0.79–0.83; p < 0.001), 0.77 (95%CI 0.75–0.79; p < 0.001), and 0.84 (95% CI 0.82–0.86; p < 0.001), respectively.

#### Small Airway Disease

Parameter D and FEV_1_/FVC were similar in accuracy for identifying >5% fSAD (c-statistic 0.76,95% CI 0.74–0.78; p < 0.001, and 0.78,95% CI 0.77–0.80; p < 0.001, respectively), whereas the c-statistic for Transition Point, Transition Distance, and FEV_1_%predicted were 0.63 (95% CI 0.61–0.64; p < 0.001), 0.59 (95% CI 0.57–0.61; p < 0.001), and 0.66 (95% CI 0.65–0.68; p < 0.001), respectively.

### Association of New Metrics with Outcomes

All three new metrics had significant associations with emphysema, small airway disease, medium size airway disease, as well as respiratory morbidity, after adjustment for age, sex, race, BMI, and scanner type for CT parameters (Table 1**)**.

**Table 1:** Associations between New Metrics and CT disease and Respiratory Morbidity.

	Parameter D		Transition Point		Transition Distance	
	Univariate regression coefficient	Multivariable regression coefficient*	Univariate regression coefficient	Multivariable regression coefficient*	Univariate regression coefficient	Multivariable regression coefficient*
% Emphysema	88.7 (84.9 to 92.6)	82.1 (78.0 to 86.2)	−0.34 (−0.36 to −0.33)	−0.33 (−0.34 to −0.31)	−0.016 (−0.017 to −0.015)	−0.014 (−0.015 to −0.013)
%PRM^fSAD^	176.7 (169.6 to 183.8)	153.5 (146.2 to 160.8)	−0.53 (−0.55 to −0.51)	−0.49 (−0.51 to −0.46)	−0.023 (−0.025 to −0.022)	−0.020 (−0.021 to −0.019)
WallArea%	21.2 (19.4 to 23.1)	30.1 (28.3 to 31.9)	−0.094 (−0.099 to −0.090)	−0.114 (−0.119 to −0.110)	−0.004 (−0.005 to −0.004)	−0.005 (−0.005 to −0.004)
SGRQ	157.5 (145.6 to 169.5)	206.5 (194.1 to 218.8)	−0.70 (−0.73 to −0.67)	−0.80 (−0.84 to −0.77)	−0.034 (−0.036 to −0.032)	−0.035 (−0.037 to −0.033)
mMRC	8.1 (7.4 to 8.9)	11.2 (10.4 to 12.0)	−0.042 (−0.044 to −0.040)	−0.045 (−0.047 to −0.043)	−0.002 (−0.002 to −0.002)	−0.002 (−0.002 to −0.002)

^*^Adjusted for age, sex, race, and BMI as well as scanner type in the case of CT parameters.

All p values were < 0.001.

PRM = Parametric response mapping. fSAD = Functional small airway disease. SQRQ = St. George’s Respiratory Questionnaire. MMRC = Modified Medical Research Council Dyspnea Scale.

### Association with Mortality

We had follow-up data on 7294 participants for a median (interquartile range, IQR) of 6.6 (5.8 to 7.3) years. 993 (12.0%) participants died on follow-up. Follow-up data was available in 4843 (88%) of participants in whom Parameter D could be calculated. When subjects were categorized into quartiles of Parameter D, the higher two quartiles (≥−0.082 and −0.113 to −0.082) were associated with greater mortality compared with the lowest quartile (≤−0.142), unadjusted hazards ratio, HR 4.47,95% CI 3.42–5.85; p < 0.001 and 1.41,95% CI 1.03–1.93; p = 0.031, respectively. After adjustment for age, sex, race and body-mass-index (BMI), only the highest quartile was significantly associated with mortality compared to the lowest quartile (adjusted HR 3.22,95% CI 2.42–4.27; p < 0.001). Of note, there were a substantial number of participants in the highest quartile of Parameter D who were categorized as GOLD 0 and 1 by traditional criteria (1.8% and 33.7%, respectively). On the other hand, 90.3% and 99.3% of GOLD 3 and 4 in whom Parameter D could be measured were comprised of participants in the highest quartile of Parameter D. Mortality data for Transition Point and Transition Distance are shown in the Supplement.

## Mild Disease

In the second stage of analysis, we focused on 4870 participants with GOLD stage 0 and 1. Mean age was 57.5 (SD 8.6) years, and the subset was comprised of 46.4% females, and 37.7% African Americans. Both the Transition point and Transition Distance could be calculated in 4686 (96.2%) of expiratory curves whereas Parameter D could be calculated in 3930 (80.7%). 760/4870 (15.6%) had airflow obstruction by traditional GOLD criteria and 445/4870 (9.1%) using the LLN criteria for FEV_1_/FVC. 873/3930 (17.9%), 721/4686 (14.8%), and 788/4612 (16.2%), respectively had airflow obstruction per Parameter D, Transition Point, and Transition Distance, respectively.

### Comparison of Traditional Criteria and Parameter D

**Figure 3:**
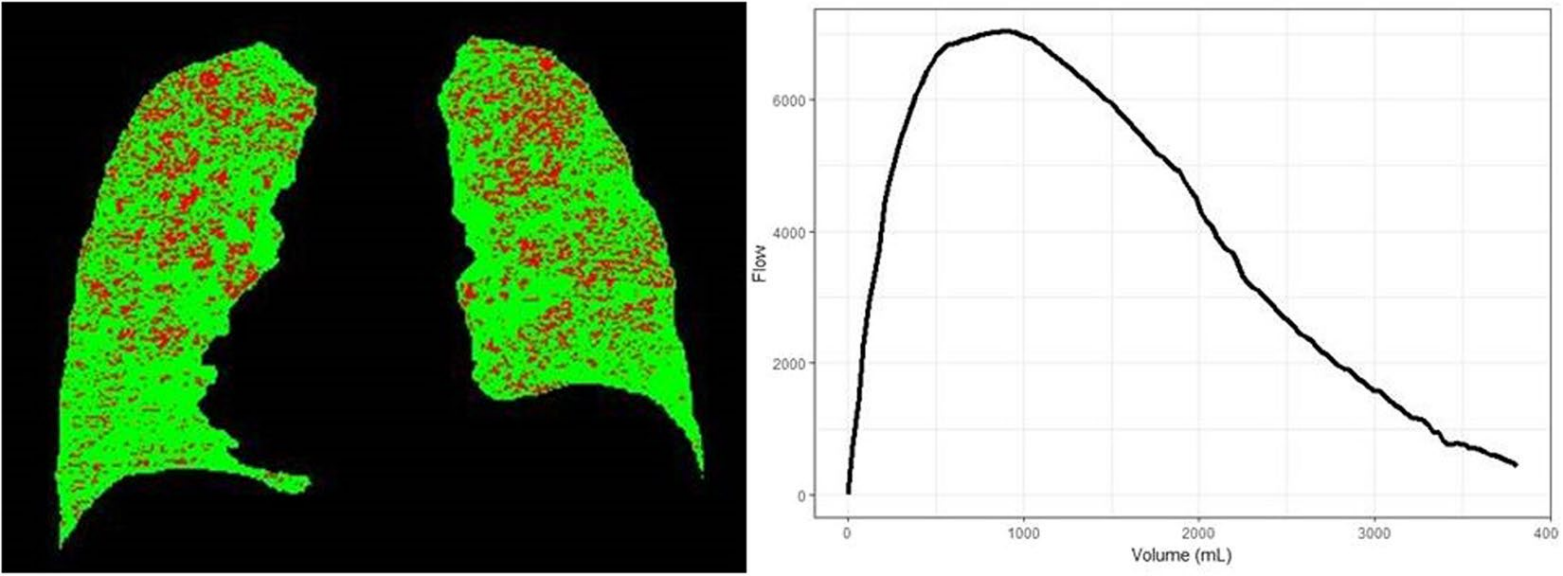
Representative images for a 54 year old African American male with a 34 pack-year smoking history who had significant symptom burden, with mMRC score of 3, and SGRQ score of 48. His lung function by traditional criteria was normal with FEV_1_/FVC of 0.72, and FEV_1%_predicted of 100.1%. Flow volume curve appears normal, however Parameter D was −0.08, which is abnormal. Computed tomography revealed 0.5% emphysema and 25% fSAD (highlighted in red). mMRC = Modified Medical Research Council Dyspnea Scale. SQRQ = St. George’s Respiratory Questionnaire. FEV_1_ = Forced Expiratory Volume in the first second. FVC = Forced Vital Capacity. fSAD = Functional small airway disease.

Table 2 shows a comparison of participants concordant and discordant for abnormality by both FEV_1_/FVC < 0.70 and Parameter D. Parameter D identified an additional 9.5% of participants with mild or non-recognized disease as abnormal, and this proportion was 11.8% where Parameter D was calculable. Compared with participants who were concordant normal, these discordant cases positive by Parameter D alone were similar in age but with higher FEV_1_ and FVC as well as CT TLC and FRC, but had higher CT measures of emphysema, functional small airway disease as well as segmental bronchial wall thickness. These relationships held true after adjusting for age, sex, race, BMI, and CT scanner type (Table 3). Of the 465 discordant cases positive by Parameter D alone, more subjects had emphysema >5% (20.0% vs. 8.9%; p < 0.001) and PRM^fSAD^ > 15% (43.4% vs. 26.5%; p < 0.001), compared with concordant normals. Of those positive by Parameter D alone, 115 (24.7%) and 91 (19.6%) had substantial symptoms as evidenced by St. George’s Respiratory Questionnaire (SGRQ) score >25 and modified Medical Research Council (mMRC) dyspnea score >2, respectively. Figure 3 shows a representative subject not detected by traditional criteria but had abnormal Parameter D.

**Table 2:** Comparison of demographics, imaging and respiratory morbidity between concordant and discordant groups by Parameter D and FEV1/FVC < 0.70^#^

	“Normal” controls (Both FEV_1_/FVC and Parameter D negative) (n = 2896)	COPD (Both FEV_1_/FVC and Parameter D positive) (n = 408)	FEV1/FVC Discordant (FEV_1_/FVC positive and Parameter D negative) (n = 161)	Parameter D Discordant (Parameter D positive and FEV_1_/FVC negative) (n = 465)
**Demographics**				
Age (years)	56.5 (8.2)	61.4 (8.7)^‡^	62.6 (9.3)^‡^	56.8 (8.5)
Sex (%Males)^‡^	51.8	63.5	49.1	65.8
Race (%White)^‡^	57.9	82.1	73.9	71.4
BMI (kg/m^2^)	29.0 (5.8)	26.7 (5.1)^‡^	27.3 (4.5)**	27.6 (5.2)^‡^
Pack-years	37.0 (20.1)	46.9 (25.8)^‡^	43.1 (20.8)**	37.6 (18.2)
**Spirometry**				
FEV_1_ (L)	2.90 (0.66)	2.76 (0.68)**	2.57 (0.61)^‡^	3.06 (0.66)^‡^
FEV_1_ (%Pred)	98.1 (11.6)	90.9 (9.0)^‡^	91.6 (9.3)^‡^	95.6 (10.5)^‡^
FVC (L)	3.66 (0.85)	4.30 (1.02)^‡^	3.87 (0.89)*	4.13 (0.91)^‡^
FEV_1_/FVC	0.80 (0.05)	0.64 (0.04)^‡^	0.67 (0.03)^‡^	0.74 (0.04)^‡^
Parameter D	−0.14 (0.03)	−0.08 (0.01)^‡^	−0.12 (0.02)^‡^	−0.09 (0.01)^‡^
Transition Point	37.8 (11.6)	32.1 (10.2)^‡^	34.4 (11.3)**	35.0 (12.0)^‡^
Transition Point Distance	504.9 (217.8)	444.6 (187.7)^‡^	484.8 (215.6)	487.2 (217.2)
**CT**				
TLC (L)	5.2 (1.2)	6.4 (1.5)^‡^	5.6 (1.3)**	6.0 (1.3)^‡^
FRC (L)	2.7 (0.7)	3.4 (0.9)^‡^	3.1 (0.9)^‡^	3.2 (0.8)^‡^
%Emphysema (LAA < −910insp)	16.2 (13.3)	30.1 (15.1)^‡^	22.4 (14.9)^‡^	23.5 (15.2)^‡^
Wall Area%	59.9 (2.9)	60.2 (2.8)	60.4 (2.7)	60.4 (2.9)**
% Severe Emphysema (LAA < −950insp)	0.5 (1.1)	2.7 (3.9)^‡^	1.3 (2.3)^‡^	1.0 (1.7)^‡^
% PRM^fSAD^	11.5 (10.0)	20.9 (10.7)^‡^	18.7 (12.5)^‡^	16.1 (11.6)^‡^
**Respiratory Morbidity**				
MMRC	0.8 (1.2)	0.7 (1.1)	0.7 (1.1)	0.7 (1.2)
SGRQ	16.5 (17.7)	17.7 (17.0)	16.2 (16.9)	16.4 (17.4)

^#^COPD defined traditionally by FEV_1_/FVC < 0.70.

*p < 0.05 compared to “normal” controls.

**p < 0.01 compared to “normal” controls.

^‡^p < 0.001 compared to “normal” controls.

BMI = Body Mass Index. FEV_1_ = Forced Expiratory Volume in the first second. FVC = Forced Vital Capacity. TLC = Total Lung Capacity on computed tomography. FRC = Functional Residual Capacity on computed tomography. %LAA < 910insp = %Low Attenuation Area below a threshold of −910 Hounsfield Units at end inspiration. Wallarea% = Bronchial wall area at segmental level. PRM = Parametric response mapping. fSAD = Functional small airway disease.

MMRC = Modified Medical Research Council Dyspnea Scale. SQRQ = St. George’s Respiratory Questionnaire.

**Table 3:** Odds Ratios of COPD diagnostic criteria for predicting imaging measures of COPD.

Parameter	Mild Emphysema			Emphysema			PRM^fSAD^			%Wall Area		
	Odds Ratio	95% CI	p	Odds Ratio	95% CI	p	Odds Ratio	95% CI	p	Odds Ratio	95% CI	p
COPD	3.23	2.71 to 4.07	<0.001	6.97	5.45 to 8.92	<0.001	3.98	3.20 to 4.96	<0.001	1.91	1.57 to 2.32	<0.001
FVC-COPD	1.39	1.03 to 1.88	0.03	2.41	1.74 to 3.34	<0.001	2.24	1.64 to 3.08	<0.001	1.89	1.41 to 2.53	<0.001
Parameter D-COPD	1.92	1.59 to 2.31	<0.001	2.28	1.86 to 2.79	<0.001	2.01	1.65 to 2.45	<0.001	1.61	1.34 to 1.93	<0.001

Model adjusted for age, sex, race, BMI and scanner type. All comparisons made for each group in reference with normal controls. Mild emphysema defined by %LAA < −910 HU.

PRM = Parametric response mapping. fSAD = Functional small airway disease. Wallarea% = Bronchial wall area at segmental level. CI = Confidence intervals

COPD includes subjects positive by both criteria, FEV_1_/FVC < 0.70 and Parameter D > 90^th^ percentile of normal. FVC-COPD includes subjects positive by FEV_1_/FVC < 0.70 only. Parameter D-COPD includes subjects positive by Parameter D > 90^th^ percentile of normal only.

## Discussion

In a cohort of current and former smokers, we derived new indices of airflow obstruction that identify additional subjects with structural and clinical lung disease who are deemed to be normal by traditional criteria. These new metrics are independently associated with structural lung disease on CT, as well as with dyspnea and respiratory-quality of life, and are especially useful for subjects with borderline or mild disease by traditional criteria.

Traditional spirometry criteria are simple to use and perform well in detecting more apparent disease, but do miss a number of mild cases who might benefit from intervention. Parameter D identified a substantial number of additional asymptomatic and symptomatic patients who would otherwise be missed by the traditional criteria. The new metrics can also be used to identify subjects with mild disease with a high risk of mortality. Results from two large cohort studies have found that approximately one-half of participants without overt airflow obstruction using traditional criteria have substantial respiratory morbidity and structural changes on CT^2,3^. These symptomatic smokers are also at increased risk of greater lung function decline and developing overt airflow obstruction on follow-up^4^. These findings point to the lack of sensitivity of traditional spirometric measures in detecting mild disease. Our findings have important clinical implications. The utility of Parameter D lies not in supplanting existing spirometry measures, but in being able to find additional cases and hence increasing sensitivity for case finding. These new metrics can be easily adapted into commercially available spirometry software without any change in testing procedures to provide additional outputs that can help inform the likelihood of airflow obstruction in borderline cases. In cases where volume-time curves or flow-volume curves are sampled at frequencies different from our study, the curves can be resampled at the same rate as in our study and the codes shown in the Supplement applied.

Spirometric measures of airflow obstruction have mostly relied on utilizing fixed portions of the expiratory flow-volume curves, and have not seen major changes in decades. Although measures of FEV_1_/FVC and FEV_1_%predicted have stood the test of time, multiple recent studies suggest that these measures do not detect mild abnormalities detected by other methods such as plethysmography, diffusing capacity of the lung for carbon monoxide, and structural measures of disease on computed tomography^23–25^. Spirometric measures of small airway disease such as FEF_25–75%_ suffer from wide variability, and others such as FEV_3_/FVC are also dependent on fixed segments of the expiratory curve^26^. Although qualitative assessment of expiratory curves has long been used to assess airflow obstruction, these changes are not readily apparent until the disease is far advanced. Parameter D, by partly representing the slow exponential decay in volume over the later part of the volume-time curve, is likely a reflection of small airway involvement and changes in elastic recoil of the lung. Using image matching, we show that Parameter D is strongly associated with PRM^fSAD^, a measure of non-emphysematous gas trapping^18^.

Since Salztman *et al*. tied the spirographic “kink”^27^, the angle between the steep and shallow parts of the expiratory curve, to emphysema and diffusing capacity of carbon monoxide (DLCO), multiple studies have quantified this angle. Kapp and colleagues found that the angle was progressively more acute with worsening airflow obstruction^14^. Topalovic *et al*. identified a threshold of 131^◦^ for the angle of collapse with a high specificity for emphysema albeit with poor sensitivity^28^. Wang and group used angle of collapse ≤ 137° to differentiate asthma-COPD overlap subjects with significant emphysema^15^. Dominelli and group calculated the slope-ratio of the middle 20–80% of the expiratory curve to quantify its concavity, and to differentiate mild COPD from healthy older subjects^29^. The study included only symptomatic COPD and included elderly adults. Other measures have included the flow ratio expressed as a percentage of the instantaneous flow at 75 percent of the expired vital capacity (FR75)^8^, quantification of the curvature (kmax index)^9^, and shape factors at 25% and 50% of exhalation^10^. Most of these studies had small number of subjects and tied these metrics to FEV_1_. Two studies compared the new metrics with emphysema but did not have measurement of small airway disease^15,28^. By testing our metrics against measures of structural airway and parenchymal disease on CT as well as with respiratory morbidity and mortality, we extend the literature by introducing robust metrics that add to the traditional measurements.

Our study has several strengths. We analyzed data from a large cohort of current and former smokers at risk for airflow obstruction, who were extensively phenotyped with spirometry and CT that were subject to stringent quality control. The new spirometry metrics were tested against structural lung disease on CT. We included a large number of African Americans, as well as women. We also note several limitations. The expiratory CT scans were acquired at FRC whereas the expiratory effort during spirometry ends in residual volume. However, by validating these forced expiratory measures in the possibly less sensitive tidal breath scans in COPDGene, our validation measures are likely stronger. We acknowledge that Parameter D could not be assessed in those with very severe disease (Supplemental Table 3), but this metric is likely to be more useful as an additive metric in those with mild disease. Mortality models were not adjusted for FEV_1_ as Parameter D and FEV_1_ are correlated due to the nature of measurements. However, we show that there is significant discordance between quartiles of Parameter D and GOLD stages, thus enabling identification of smokers in milder GOLD stages who have poor outcomes. Lastly, we analyzed subjects who were current and former smokers, and hence the results need to be validated in other populations at varying risk for airflow obstruction.

## Conclusions

In summary, we developed several new and easily applicable metrics of mild airflow obstruction that in combination with existing spirometry criteria have the potential to identify additional subjects with structural lung disease, and respiratory morbidity.

## Electronic supplementary material

Supplement

## References

[1] Vestbo J (2013). Global strategy for the diagnosis, management, and prevention of chronic obstructive pulmonary disease: GOLD executive summary. Am J Respir Crit Care Med.

[2] Regan EA (2015). Clinical and Radiologic Disease in Smokers With Normal Spirometry. JAMA Intern Med.

[3] Woodruff PG (2016). Clinical Significance of Symptoms in Smokers with Preserved Pulmonary Function. N Engl J Med.

[4] Lindberg A (2007). Decline in FEV1 in relation to incident chronic obstructive pulmonary disease in a cohort with respiratory symptoms. Copd.

[5] Bhatt, S. P. *et al*. CT-derived Biomechanical Metrics Improve Agreement Between Spirometry and Emphysema. *Acad Radiol* (2016). 10.1016/j.acra.2016.02.002 PMC5026854 27055745

[6] Tien YK, Elliott EA, Mead J (1979). Variability of the configuration of maximum expiratory flow-volume curves. J Appl Physiol Respir Environ Exerc Physiol.

[7] O’Donnell CR (1987). Accuracy of spirometric and flow-volume indices obtained by digitizing volume-time tracings. Am Rev Respir Dis.

[8] O’Donnell CR, Rose RM (1990). The flow-ratio index. An approach for measuring the influence of age and cigarette smoking on maximum expiratory flow-volume curve configuration. Chest.

[9] Zheng CJ, Adams AB, McGrail MP, Marini JJ, Greaves IA (2006). A proposed curvilinearity index for quantifying airflow obstruction. Respir Care.

[10] Ohwada A, Takahashi K (2012). Concave pattern of a maximal expiratory flow-volume curve: a sign of airflow limitation in adult bronchial asthma. Pulm Med.

[11] Neve V, Edme JL, Baquet G, Matran R (2015). Reference ranges for shape indices of the flow-volume loop of healthy children. Pediatr Pulmonol.

[12] Li, H., Liu, C., Zhang, Y. & Xiao, W. The Concave Shape of the Forced Expiratory Flow-Volume Curve in 3 Seconds Is a Practical Surrogate of FEV1/FVC for the Diagnosis of Airway Limitation in Inadequate Spirometry. *Respir Care* (2016). 10.4187/respcare.05016 27999150

[13] Varga J (2016). Relation of concavity in the expiratory flow-volume loop to dynamic hyperinflation during exercise in COPD. Respir Physiol Neurobiol.

[14] Kapp MC, Schachter EN, Beck GJ, Maunder LR, Witek TJ (1988). The shape of the maximum expiratory flow volume curve. Chest.

[15] Wang W, Xie M, Dou S, Cui L, Xiao W (2016). Computer quantification of “angle of collapse” on maximum expiratory flow volume curve for diagnosing asthma-COPD overlap syndrome. Int J Chron Obstruct Pulmon Dis.

[16] Regan EA (2010). Genetic epidemiology of COPD (COPDGene) study design. Copd.

[17] Pellegrino R (2005). Interpretative strategies for lung function tests. Eur Respir J.

[18] Galban CJ (2012). Computed tomography-based biomarker provides unique signature for diagnosis of COPD phenotypes and disease progression. Nature medicine.

[19] Bhatt SP (2014). FEV(1)/FEV(6) to diagnose airflow obstruction. Comparisons with computed tomography and morbidity indices. Ann Am Thorac Soc.

[20] Vogelmeier CF (2017). Global Strategy for the Diagnosis, Management, and Prevention of Chronic Obstructive Lung Disease 2017 Report. GOLD Executive Summary. Am J Respir Crit Care Med.

[21] Wan ES (2011). Clinical and radiographic predictors of GOLD-unclassified smokers in the COPDGene study. Am J Respir Crit Care Med.

[22] Hankinson JL, Odencrantz JR, Fedan KB (1999). Spirometric reference values from a sample of the general U.S. population. Am J Respir Crit Care Med.

[23] Topalovic M (2015). Airways resistance and specific conductance for the diagnosis of obstructive airways diseases. Respir Res.

[24] Harvey BG (2015). Risk of COPD with obstruction in active smokers with normal spirometry and reduced diffusion capacity. Eur Respir J.

[25] Bhatt SP (2014). Comparison of spirometric thresholds in diagnosing smoking-related airflow obstruction. Thorax.

[26] Dilektasli AG (2016). A Novel Spirometric Measure Identifies Mild COPD Unidentified by Standard Criteria. Chest.

[27] Saltzman HP, Ciulla EM, Kuperman AS (1976). The spirographic “kink”. A sign of emphysema. Chest.

[28] Topalovic M (2013). Computer quantification of airway collapse on forced expiration to predict the presence of emphysema. Respir Res.

[29] Dominelli PB (2015). Quantifying the shape of the maximal expiratory flow-volume curve in mild COPD. Respir Physiol Neurobiol.

