# Supplementary material for: New Spirometry Indices for Detecting Mild Airflow Obstruction
Source: Sci Rep. 2018 Nov 30;8:17484. doi: 10.1038/s41598-018-35930-2 (PMC6269456; doi:10.1038/s41598-018-35930-2)
Supplement: Supplementary file 1 — Supplement [file 41598_2018_35930_MOESM1_ESM.docx]

**Supplement: New Spirometry Indices for Detecting Mild Airflow Obstruction**

Surya P. Bhatt, M.D, M.S.P.H.,^1,2^ Nirav R. Bhakta, M.D.,^3^ Carla G. Wilson, M.S.^4^, Christopher B. Cooper, M.D.,^5^ Igor Barjaktarevic, M.D.,^5^ Sandeep Bodduluri, Ph.D.,^1,2^ Young-il Kim, Ph.D.,^1,6^ Michael Eberlein, M.D., Ph.D.,^7^ Prescott G. Woodruff, M.D., M.P.H.,^3^ Frank C. Sciurba, M.D.,^8^ Peter J. Castaldi, M.D.,^9^ MeiLan K. Han, M.D.,^10^ Mark T. Dransfield, M.D.,^1,2^ Arie Nakhmani, Ph.D.^11^

^1^Division of Pulmonary, Allergy and Critical Care Medicine and Lung Health Center, University of Alabama at Birmingham, Birmingham, AL 35294; ^2^UAB Lung Imaging Core, University of Alabama at Birmingham, Birmingham, AL 35294; ^3^Division of Pulmonary, Critical Care, Allergy and Sleep Medicine, University California San Francisco, San Francisco, CA 94143; ^4^Department of Biostatistics and Bioinformatics, National Jewish Health, Denver, CO 80206; ^5^Division of Pulmonary and Critical Care Medicine, David Geffen School of Medicine at the University California Los Angeles, Los Angeles, CA 90095; ^6^Department of Preventive Medicine, University of Alabama at Birmingham, Birmingham, AL 35294; ^7^Division of Pulmonary, Critical Care and Occupational Medicine, University of Iowa Hospital, Iowa City, IA 52242; ^8^Division of Pulmonary, Allergy and Critical Care Medicine, University of Pittsburgh, Pittsburgh, PA 15213; ^9^Channing Division of Network Medicine, Brigham and Women's Hospital, Boston, MA 02115; ^10^Division of Pulmonary and Critical Care Medicine, University of Michigan, Ann Arbor, MI 48109; ^11^Department of Electrical and Computer Engineering, University of Alabama at Birmingham, Birmingham, AL 35294.

**Methods**

**Imaging**

Volumetric CT scans were obtained at maximal inspiration (total lung capacity, TLC) and end-tidal expiration (functional residual capacity, FRC). Emphysema and gas trapping were quantified using 3D Slicer software ([www.airwayinspector.org](http://www.airwayinspector.org)), and Pulmonary Workstation 2 (VIDA Diagnostics, Coralville, IA, USA) was used to measure airway dimensions.^1^ Mild emphysema was quantified by using the percentage of lung volume at TLC with attenuation less than -910 Hounsfield Units (HU) (low attenuation area, %LAA910_insp_), and severe emphysema by %LAA<-950 HU.^1^ We quantified gas trapping as the percentage of lung volume at end expiration with attenuation less than -856 HU.^1^ We used Wall area percentage of segmental airways (Wall area pct) to quantify airway disease.^1^ In addition, we used parametric response mapping to match inspiratory and expiratory images voxel-to-voxel, and calculated the percentage of non-emphysematous gas trapping, or functional small airways disease (PRM^fSAD^), a measure of small airways disease.^2^

**Respiratory Morbidity Measures**

St George’s Respiratory Questionnaire (SGRQ) score was used to assess respiratory quality of life,^3^ and dyspnea was measured using the modified Medical Research Council (mMRC) dyspnea score.^4^

**Spirometry Quality Control and Selection**

All subjects participating in the COPDGene study, a large multicenter cohort, were included. Enrollment criteria included current and former smokers aged 45 to 80 years, of Non-Hispanic White and African American races. All participants were assessed using pre and post bronchodilator spirometry using the ndd Easy-One spirometer according to the American Thoracic Society (ATS) criteria. Pre bronchodilator spirometry was followed by administration of 2 puffs of albuterol HFA using appropriate spacers such as Aerochamber® (Monaghan Medical Corporation, Plattsburgh, NY). Post-bronchodilator spirometry was performed 15-20 minutes post albuterol administration. We used post bronchodilator values for all analyses.

A stringent quality control (QC) process was followed for all spirometry measurements. In the first stage, QC was automated, and the three best efforts for pre- and post- bronchodilator efforts for each participant were selected and stored at the clinical site. These data were transferred electronically to the Data Coordinating Center for the Spirometry Core. In the second stage, all spirometry measurements were reviewed at the Spirometry Core and graded by an automated QC software according to the ATS criteria as follows: Grade 4: efforts fully met ATS criteria, reproducible to within 50 ml, Grade 3: efforts fully met ATS criteria, reproducible between 50 and 100 ml, Grade 2: efforts fully met ATS criteria, reproducible between 100 and 150 ml, Grade 1: efforts partly met ATS criteria and/or reproducible between 150 and 200 ml, and Grade 0: efforts failed to meet ATS criteria and/or reproducible greater than 200 ml.^5^ Primary QC grades between 4 and 2 were considered passing grade, and those with either 1 or 0 were of failing grade. Of note, the mean primary grade for the cohort was between 3.1 and 3.5 for post bronchodilator FEV_1_ and FVC. At each clinical site, those spirometries that failed the automated QC were repeated when possible. Those spirometries that were of a failing grade on automated QC and could not be repeated at the clinical site were reviewed in a third stage by the Spirometry Core Review Committee. This Committee also assessed technical issues such as spirometer malfunctioning and zero-flow errors to evaluate whether spirometries met the standard for inclusion. The effort with the highest sum of FEV_1_ and FVC were chosen for analysis.

**Physiologic Basis of New Metrics:**

On forced exhalation during spirometric maneuvers, the flow-volume curve has an ascending part culminating in a peak, and a subsequent descending part where flow slows down with progressive emptying of the lung (**Supplemental Figure 1**). The transition from the steep to the shallow slope during the descending part has been variously termed the Transition Point, or the spirographic kink.^6^ The initial peak of the flow-volume curve depends on subject effort, as well as the patency of central airways. The latter portion of the descending curve is relatively effort-independent and the slope of this portion of the curve depends on small airway resistance and elastic recoil of the lung. There are multiple physiological factors that affect this change in slope. The equal pressure point (EPP) is the point at which pressure inside the airway is equal to the pressure external to the airways (intra-pleural pressure). In healthy lungs, the EPP usually occurs in cartilaginous airways as a result of sufficient alveolar driving pressure and only a gradual drop in pressure due to minimal airway resistance. With increase in airway resistance or loss of small airways, the pressure drop becomes much steeper, and the EPP moves toward the alveoli and may be reached in the thin-walled bronchioles, causing airway narrowing or collapse and the typical pressure-dependent collapse in the flow-volume curve. The loss of lung elastic recoil that occurs due to emphysema-related alveolar septal reduction also contributes to migration of the EPP towards the alveoli, by reducing the alveolar driving pressure and thereby also reducing the pressure drop required for airway pressure to equal intrapleural pressure; loss of elastic recoil also reduces the connective tissue support that surrounds and tethers thin-walled bronchioles. In addition, heterogeneity of disease distribution in emphysema results in rapid emptying of relatively normal lung regions and slower emptying of diseased regions. A combination of movement of the EPP, loss of airway support, and heterogeneous emptying can break the descending slope into two, an initial steep part (S1) and a later shallow and prolonged part (S2) at the Transition Point (**Supplemental Figure 1**). In addition, with increasing disease severity, the breaking point is likely to move closer to TLC, and decrease the volume along the X axis to reach the breaking point (Transition Distance). Using advanced computational tools, we analyzed the individual data points in the flow-volume and volume-time curves, and developed the following novel metrics to quantify important transition points and contours in expiratory curves.

# **
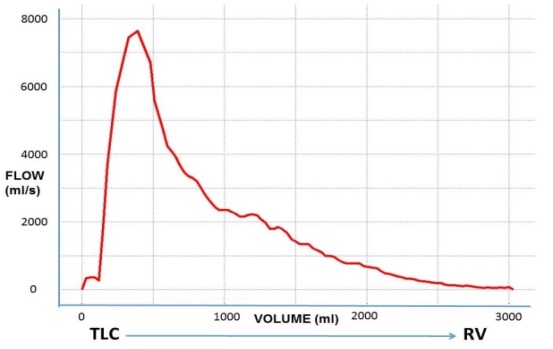
**

# **Supplemental Figure 1: Early and late descending slopes of the expiratory Flow-Volume curve.**

# **Derivation of New Metrics:**

All in-house scripts were developed using MATLAB (The MathWorks, Inc., Natick, MA). Each spirometry effort results in two standard spirometry curves on exhalation: Volume-Time curve: Volume V[ml] as a function of time t[ms], and the Flow-Volume curve: Flow F[ml/ms] as a function of Volume V[ml]). Flow is sampled once per 60[ms], and Volume is sampled once per 30[ml]. In our approach, in addition to extraction of various curve parameters (e.g., the transition point), we fit the entire curve to a nonlinear mathematical model with minimal number of parameters. If the fit is good, the parameters would include all the available information about the curve, because the entire curve could be reconstructed using those parameters. This also means that all known and future spirometry measures could be computed from those parameters.

# **Shape of the Volume-Time curve and Computation of Volume-Time curve fitting parameters**

**Supplemental Figure 2: Cropping the curve to normalize the initial exhalation time**

Before the model definition and computation of its parameters, all curves were converted into MATLAB database and cropped to start from the first sample of exhalation (e.g., see **Supplemental Figure 2**). The first sample of exhalation was defined as the first sample above 20ml. Also, for each patient only the best out of three curves was selected to extract the parameters. The best curve was selected with the maximal FEV_1_+FVC, when FVC and FEV_1_ were automatically computed from the curve using our in-house MATLAB code.

For the volume-time curve, we used the Levenberg-Marquardt algorithm^7^ to fit the following model to the curve:

$$V_{estimated}=Ae^{Bt}+Ce^{Dt}$$

where $A,B,C,D$ are the parameters found by the function fitting optimization process. The first exponent is almost flat and describes the rising slope of a breath volume increase closer to the end of the exhalation. The second exponent describes the overall volume-time curve, where Parameter D describes the rate of volume increase. To visualize the impact of *D*, **Supplemental Figure 3** shows the plot of $V_{estimated}$ with constant parameters $A,B,C$ and changing *D*.


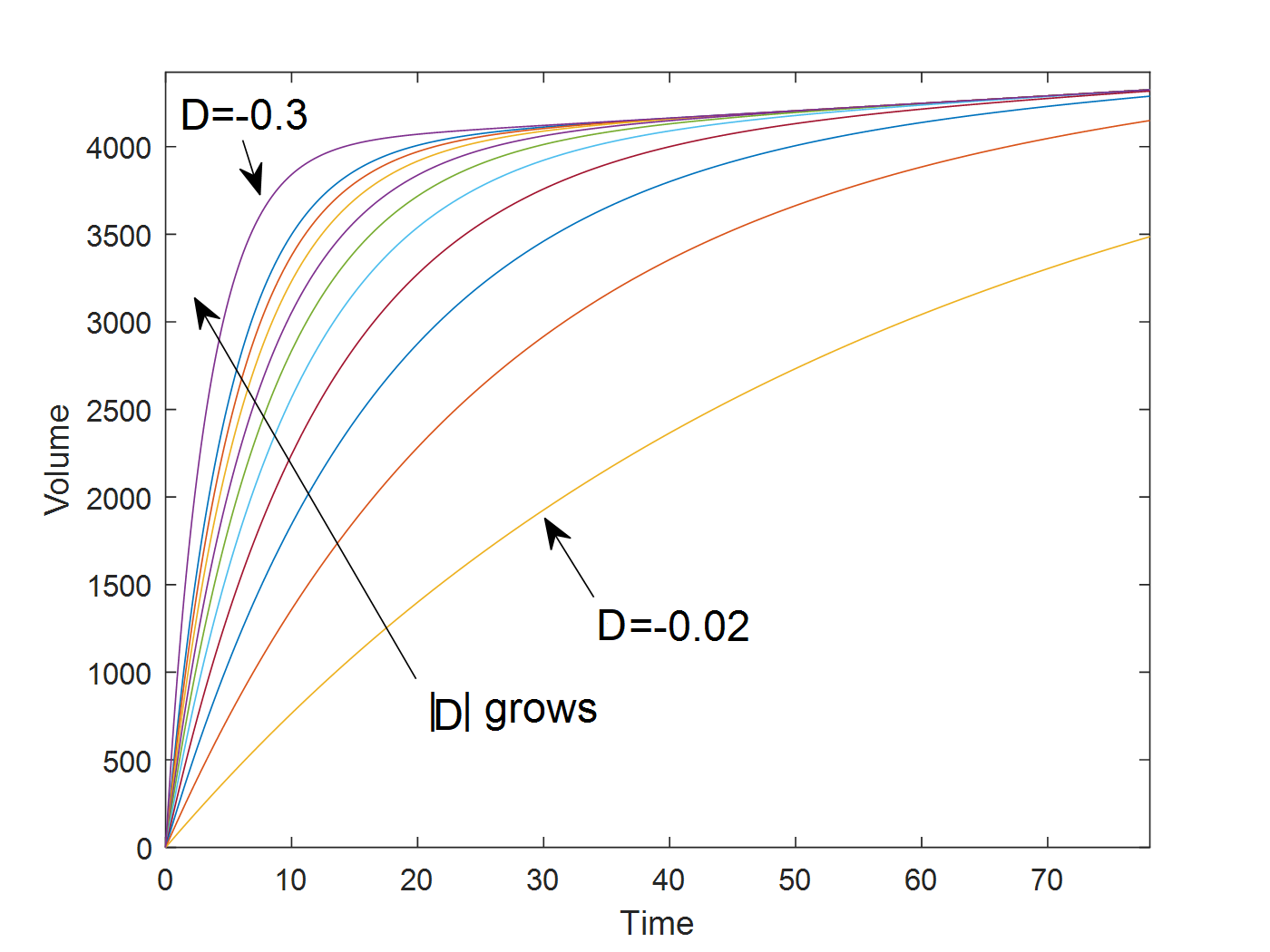


**Supplemental Figure 3: The effect of changing parameter D in the model.**

The parameters $A,B,C,D$ were computed by minimizing $J=\left\| V_{measured}-Ae^{Bt}-Ce^{Dt} \right\|$ cost function. The examples of such a function fit are given in **Supplemental Figure 4**. The fitting was done using MATLAB fit function with a double exponential model:

[xData, yData] = prepareCurveData( time, volume );

ft = fittype( 'exp2' );

opts = fitoptions( 'Method', 'NonlinearLeastSquares' );

opts.Display = 'Off';

[fitresult, gof] = fit( xData, yData, ft, opts );

plot( fitresult, xData, yData );

**Supplemental Figure 4: Model fitting. Left: Normal control volume-time curve; Right: GOLD Stage 2 COPD volume-time curve**

**Computation of the Transition Point**

In the majority of the Flow-Volume curves, there is a point where the slope significantly changes. We refer to this feature point as the Transition Point. To identify this point mathematically, it was defined by fitting a piecewise function with two linear segments to the flow curve, where the data before the point of maximum was ignored (see **Supplemental Figure 5**). A nonlinear least-squares algorithm was used to find the optimal fit parameters of the curve $\left( x_{1},y_{1} \right), \left( x_{2},y_{2} \right), (x_{3},y_{3})$. The transition breaking point is defined as $x_{2}$. The fit could be computed in MATLAB using fit function with a custom piecewise linear model:

ft = fittype( 'piecewiseLine( x, a, b, c, k )' );

x1 = 1; x3 = numel(yData); x2 = 0.5*(x1+x3);

y1 = yData(x1); y3 = 0; y2 = 0.5*(y1+y3);

opts = fitoptions( 'Method','NonlinearLeastSquares','StartPoint', ...

[(y1-y2)/(x1-x2),(y1-y2)/(x1-x2)*(-x1)+y1, (y2-y3)/(x2-x3),x2] );

f = fit( xData, yData, ft, opts);

x2 = f.k;

where,

function y = piecewiseLine(x,a,b,c,k)

y = zeros(size(x));

for i = 1:length(x)

if x(i) < k

y(i) = a.*x(i) + b;

else

y(i) = c.*x(i) + a*k-c*k+b;

end

end

end


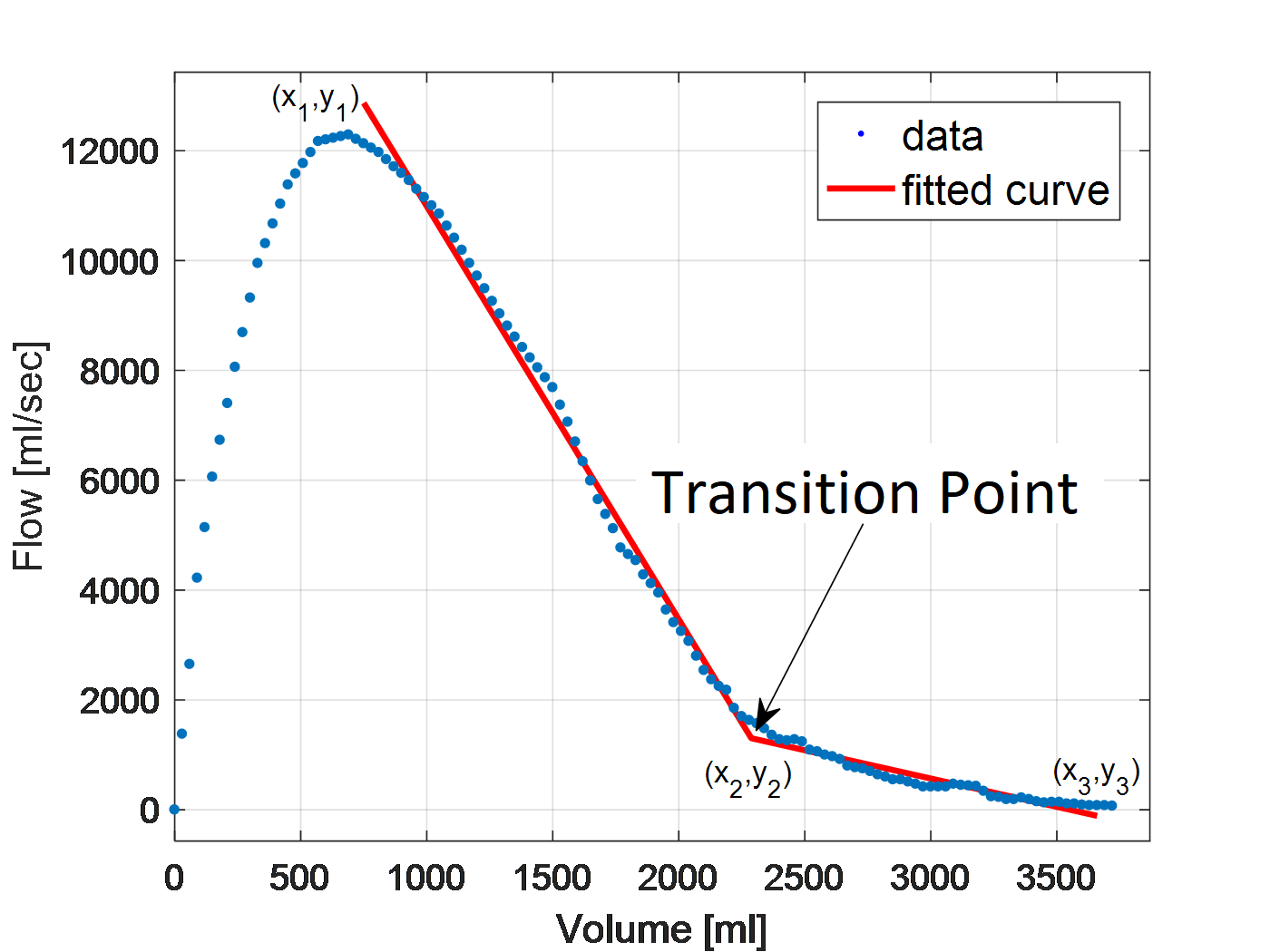


**Supplemental Figure 5: Computation of a transition breaking point feature**

**Computation of the Transition Distance**

On the Flow-Volume curve, the distance to the breaking point where the steep descending slope switches to the shallower part of the descending slope was computed. To define this distance uniquely, an inverted parabola $\alpha V^{2}+\beta V+\gamma$ was fitted around the peak point using *N* first samples and least squares minimization algorithm (see **Supplemental Figure 6**).

The following algorithm was used to define the new volume distance measure:

$N=10$

Fit the parabola to the first N samples

While the goodness of least squares fit is $R^{2}>0.96$

$N = N+1$

Fit the parabola to the first $N$ samples

end

Return $N, \alpha,\beta,\gamma$

The distance is defined as: $Distance=N\cdot30ml+\frac{\beta}{2\alpha}$.

Note that $\frac{-\beta}{2\alpha}$ is the location of maximum point on the inverted parabola (**Supplemental Figure 6**).

The breaking point was defined as the latest (in volume) sample that still provided a goodness of fit of R^2^>0.96. The change in volume (ml) on the X-axis from the peak of the parabola to the breaking point was termed the Transition Distance (**Supplemental Figure 6**). As the EPP moves peripherally with greater small airway disease, this transition distance is expected to move closer to the TLC or “zero” on the X-axis.


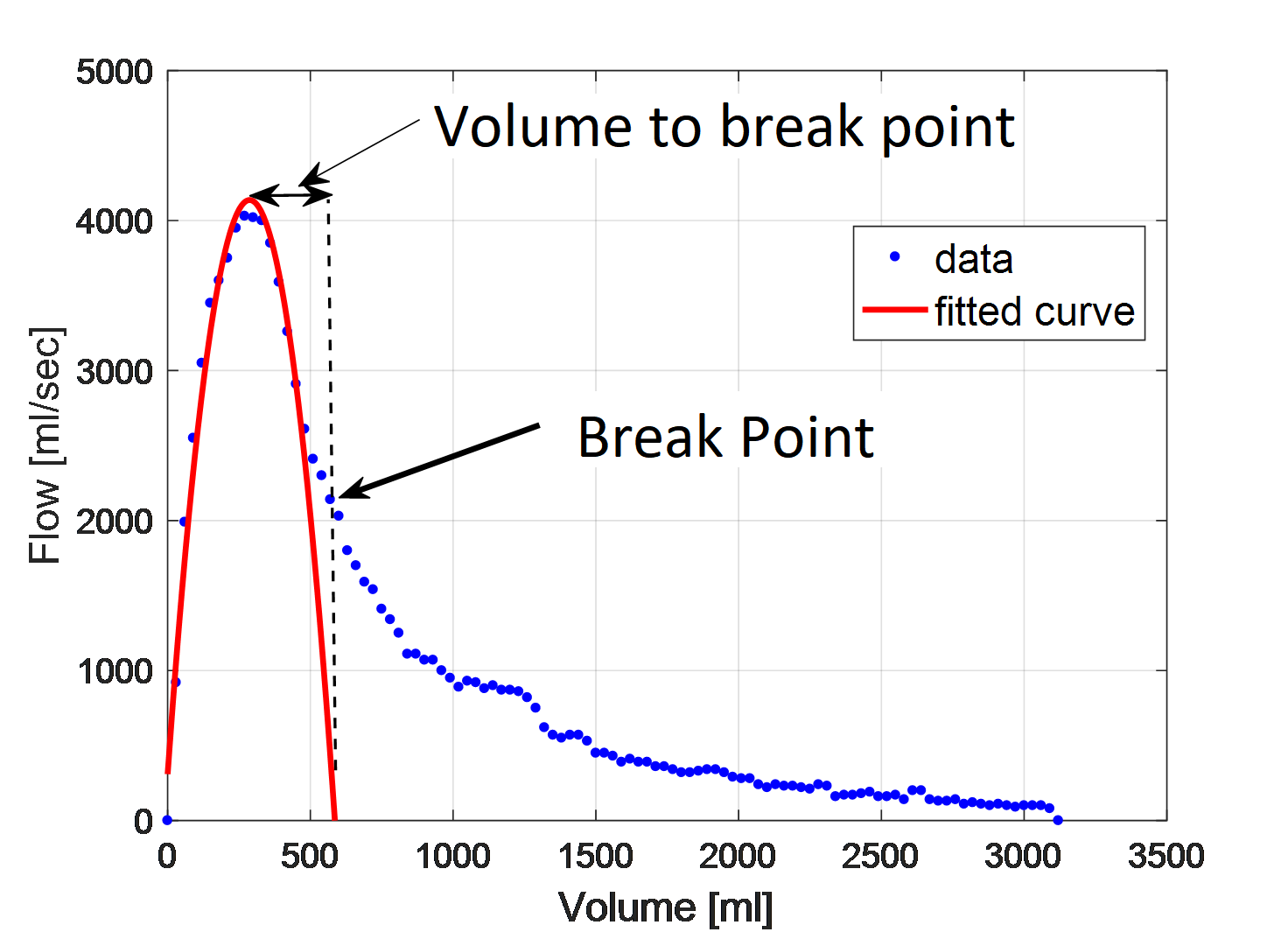


**Supplemental Figure 6: Computation of the breaking point and the change of volume from the maximum to the breaking point (Transition Distance)**

**Supplemental Figure 7: CONSORT Diagram**

**Additional Results**

**Transition Point, Transition Distance and Mortality**

Transition Point and Transition Distance were less strongly associated than Parameter D with COPD measures and outcomes. The highest Transition Point quartile was associated with greater mortality risk compared with the lowest quartile on univariate analysis (unadjusted HR 5.22,95%CI 4.21-6.23;p<0.001), and after adjustment for age, sex, race and BMI (adjusted HR 4.34,95%CI 3.52-5.36;p<0.001). The highest Transition Distance quartile was also associated with greater mortality risk compared with the lowest quartile on univariate analysis (unadjusted HR 2.70,95%CI 2.08-3.52;p<0.001), and after adjustment for age, sex, race and BMI (adjusted HR 2.55,95%CI 1.95-3.33;p<0.001).

**Supplemental Table 1: Comparison of demographics, imaging and respiratory morbidity between concordant and discordant groups by Parameter D and FEV_1_/FVC<LLN^¥^**

|  | **“Normal” controls** (Both FEV_1_/FVC and Parameter D negative) (n=2985) | **COPD**  (Both FEV_1_/FVC and Parameter D positive) (n =250) | **FEV1/FVC Discordant**  (FEV_1_/FVC positive and Parameter D negative)  (n = 72) | **Parameter D Discordant**  (Parameter D positive and FEV_1_/FVC negative)  (n = 623) |
| --- | --- | --- | --- | --- |
| Demographics |  |  |  |  |
| Age (years) | 56.9 (8.3) | 59.6 (8.7) ‡ | 56.7 (8.6) | 58.7 (8.9) ‡ |
| Sex (%Males) ‡ | 52.0 | 50.0 | 34.7 | 70.6 |
| Race (%Caucasian) ‡ | 58.8 | 75.2 | 55.6 | 76.9 |
| BMI (kg/m^2^) | 29.0 (5.8) | 26.5 (5.4) ‡ | 27.6 (5.6) | 27.4 (5.0) ‡ |
| Pack-years | 37.3 (20.2) | 45.8 (24.6) ‡ | 38.4 (19.1) | 40.4 (21.5)** |
| Spirometry |  |  |  |  |
| FEV_1_ (L) | 2.89 (0.67) | 2.63 (0.65) ‡ | 2.51 (0.48)‡ | 3.04 (0.66) ‡ |
| FEV_1_ (%Pred) | 97.9 (11.6) | 94.8 (10.3)‡ | 91.1 (8.9)‡ | 89.8 (8.4) ‡ |
| FVC (L) | 3.66 (0.86) | 4.22 (1.03) ‡ | 3.85 (0.72) | 4.20 (0.93) ‡ |
| FEV_1_/FVC | 0.79 (0.05) | 0.62 (0.05) ‡ | 0.65 (0.04) ‡ | 0.73 (0.05) ‡ |
| Parameter D | -0.14 (0.03) | -0.08 (0.02) ‡ | -0.12 (0.02) ‡ | -0.09 (0.01) ‡ |
| Transition Point | 37.7 (11.6) | 30.8 (10.3) ‡ | 34.2 (10.2) | 34.8 (11.4) ‡ |
| Transition Point Distance | 504.2 (218.1) | 430.3 (179.2) ‡ | 489.6 (204.0) | 482.1 (212.8) |
| CT |  |  |  |  |
| TLC (L) | 5.2 (1.3) | 6.2 (1.6) ‡ | 5.2 (1.2) | 6.2 (1.3) ‡ |
| FRC (L) | 2.7 (0.7) | 3.3 (0.9) ‡ | 3.0 (0.9) * | 3.3 (0.8) ‡ |
| %LAA<-910insp | 16.6 (13.5) | 29.0 (15.3) ‡ | 16.1 (12.8) | 25.6 (15.5) ‡ |
| Wall Area% | 59.9 (2.8) | 60.4 (2.9)* | 60.7 (2.7) | 60.2 (2.9)* |
| %PRM Emphysema | 0.5 (1.1) | 3.0 (4.2) ‡ | 1.1 (2.5) * | 1.4 (2.3) ‡ |
| % PRM fSAD | 11.7 (10.2) | 20.8 (10.7) ‡ | 15.6 (13.2) * | 17.3 (11.6) ‡ |
| Respiratory Morbidity |  |  |  |  |
| MMRC | 0.8 (1.2) | 0.9 (1.2) | 1.0 (1.2) | 0.6 (1.1) |
| SGRQ | 16.4 (17.6) | 20.4 (18.5) ** | 18.9 (19.0) | 15.7 (16.5) |

^¥^COPD defined by FEV_1_/FVC less than the lower limit of normal (LLN) for age, sex, race and height.

*p <0.05 compared to “normal” controls.

** p <0.01 compared to “normal” controls.

‡p <0.001 compared to “normal” controls.

LLN = Lower limit of normal. BMI = Body Mass Index. FEV_1_ = Forced Expiratory Volume in the first second. FVC = Forced Vital Capacity. TLC = Total Lung Capacity on computed tomography. FRC = Functional Residual Capacity on computed tomography. %LAA<910insp = %Low Attenuation Area below a threshold of -910 Hounsfield Units at end inspiration. Wallarea% = Bronchial wall area at segmental level. PRM = Parametric response mapping. fSAD = Functional small airways disease. mMRC = Modified Medical Research Council Dyspnea Scale. SQRQ = St. George’s Respiratory Questionnaire.

**Supplemental Table 2: Odds Ratios of COPD diagnostic criteria by LLN^¥^ for predicting imaging measures of COPD**

|  | **Mild Emphysema** | | | **PRM Emphysema** | | | **PRM fSAD** | | | **%Wall Area** | | |
| --- | --- | --- | --- | --- | --- | --- | --- | --- | --- | --- | --- | --- |
| Parameter | Odds Ratio | 95%CI | p | Odds Ratio | 95%CI | p | Odds Ratio | 95%CI | p | Odds Ratio | 95%CI | p |
| COPD | 3.66 | 2.86 to 4.68 | <0.001 | 9.45 | 6.94 to 12.86 | <0.001 | 4.67 | 3.57 to 6.10 | <0.001 | 1.86 | 1.47 to 2.36 | <0.001 |
| FVC-COPD | 1.02 | 0.65 to 1.61 | 0.93 | 1.93 | 1.24 to 3.01 | 0.003 | 1.92 | 1.23 to 3.0 | 0.004 | 1.56 | 1.02 to 2.39 | 0.04 |
| Parameter D-COPD | 2.04 | 1.73 to 2.41 | <0.001 | 2.46 | 2.05 to 2.95 | <0.001 | 2.10 | 1.76 to 2.51 | <0.001 | 1.62 | 1.37 to 1.90 | <0.001 |

^¥^COPD defined by FEV_1_/FVC less than the lower limit of normal (LLN) for age, sex, race and height.

Model adjusted for age, sex, race , BMI and scanner type. All comparisons made for each group in reference with normal controls. Mild emphysema defined by %LAA<-910 HU.

PRM = Parametric response mapping. fSAD = Functional small airway disease. Wallarea% = Bronchial wall area at segmental level. CI = Confidence intervals

COPD includes subjects positive by both criteria, FEV_1_/FVC<Lower Limit of Normal for age, sex, race and height (LLN) and Parameter D >90^th^ percentile of normal. FVC-COPD includes subjects positive by FEV_1_/FVC<LLN only. Parameter D-COPD includes subjects positive by Parameter D >90^th^ percentile of normal only.

**Supplemental Table 3: Comparison of baseline characteristics of participants in whom Parameter could and could not be calculated**

|  | **Parameter D calculatable**  **(n=5532)** | **Parameter D non-calculatable**  **(n=2775)** |
| --- | --- | --- |
| Age (years) | 58.9 (8.9) | 62.1 (9.0) |
| Sex (%Males) ‡ | 56.2 | 51.2 |
| Race (%White) ‡ | 67.4 | 71.7 |
| Pack-years | 42.6 (23.5) | 48.3 (27.2) |
| FEV_1_ (L) | 2.53 (0.86) | 1.77 (0.92) |
| FEV_1_ (%Pred) | 85.0 (22.8) | 62.7 (28.1) |
| FVC (L) | 3.59 (0.96) | 3.03 (1.0) |
| FEV_1_/FVC | 0.70 (0.14) | 0.56 (0.18) |
| GOLD Stage |  |  |
| 0 | 81.8 | 18.2 |
| I | 74.9 | 25.1 |
| II | 57.7 | 42.3 |
| III | 37.3 | 62.7 |
| IV | 28.0 | 72.0 |
| %PRM Emphysema | 3.1 (7.2) | 9.2 (12.8) |
| % PRM fSAD | 17.8 (13.6) | 26.5 (15.2) |

FEV_1_ = Forced Expiratory Volume in the first second. FVC = Forced Vital Capacity. PRM = Parametric response mapping. fSAD = Functional small airway disease.

**IRB Approval:**

The COPDGene Study was approved by the Institutional Review Boards of all 21 participating clinical centers. Ann Arbor VA Medical Center 2014-060462 (Ann Arbor VA IRB); Baylor College of Medicine H-22209 (IRB for Baylor College of Medicine); Brigham and Women's Hospital 2007P000554 (Partners Human Research Committee); Columbia Univ. Medical Center AAAC9324 (Columbia University IRB); Duke Univ. Medical Center Pro00004464 (Duke University Health System IRB); Johns Hopkins University NA_00011524 (Johns Hopkins Medicine IRB); L.A. Biomedical Research Inst. 12756-03 (John F. Wolf, M.D. Human Subjects Committee); Michael E. DeBakey VAMC H-22202 (Institutional Review Board for Human Subject Research for Baylor College of Medicine and Affiliated Hospitals); Minneapolis VA Medical Center 4128-A (Minneapolis VA Health Care System Minnesota); Health Partners Twin Cities 07-127 (Health Partners IRB); Morehouse School of Medicine 97826 (Morehouse School of Medicine IRB); National Jewish Health 1883a (National Jewish Health IRB); Reliant Medical Group (Fallon) 1441 (Reliant Medical Group IRB); Temple University 21659 (Temple IRB); Univ. of Alabama, Birmingham F070712014 (University of Alabama at Birmingham IRB for Human Use); Univ. of California, San Diego 140070 (UCSD Human Research Protections Program); University of Iowa 200710717 (University of Iowa IRB); University of Michigan HUM00014973 (University of Michigan Medical School IRB); University of Minnesota 0801M24949 (University of Minnesota IRB Human Subjects Committee); University of Pittsburgh #07120059 (University of Pittsburgh IRB); and UTHSC at San Antonio HSC20070644H (UT Health Science Center San Antonio IRB)

**References**

1. Regan, E.A.*, et al.* Genetic epidemiology of COPD (COPDGene) study design. *Copd* **7**, 32-43 (2010).

2. Galban, C.J.*, et al.* Computed tomography-based biomarker provides unique signature for diagnosis of COPD phenotypes and disease progression. *Nature medicine* **18**, 1711-1715 (2012).

3. Jones, P.W., Quirk, F.H., Baveystock, C.M. & Littlejohns, P. A self-complete measure of health status for chronic airflow limitation. The St. George's Respiratory Questionnaire. *Am Rev Respir Dis* **145**, 1321-1327 (1992).

4. Mahler, D.A. & Wells, C.K. Evaluation of clinical methods for rating dyspnea. *Chest* **93**, 580-586 (1988).

5. Pellegrino, R.*, et al.* Interpretative strategies for lung function tests. *Eur Respir J* **26**, 948-968 (2005).

6. Saltzman, H.P., Ciulla, E.M. & Kuperman, A.S. The spirographic "kink". A sign of emphysema. *Chest* **69**, 51-55 (1976).

7. J.J., M. The Levenberg-Marquardt Algorithm: Implementation and Theory. *Numerical analysis (Proc. 7th Biennial Conf., Univ. Dundee, Dundee, 1977)* **630**, 105-116 (1978).
